# Diagnosing post-capillary hypertension in patients with left heart disease: impact of new guidelines

**DOI:** 10.1007/s00392-023-02290-5

**Published:** 2023-09-05

**Authors:** Gülmisal Güder, Theresa Reiter, Georg Fette, Moritz Hundertmark, Stefan Frantz, Caroline Morbach, Stefan Störk, Matthias Held

**Affiliations:** 1 Department of Internal Medicine I, Cardiology Division, University Hospital Würzburg, Oberdürrbacherstr. 6, 97080 Würzburg, Germany; 2 Department of Clinical Research & Epidemiology, Comprehensive Heart Failure Center, University Hospital Würzburg, Würzburg, Germany; 3 Chair of Computer Science VI, University of Würzburg, Würzburg, Germany; 4 Department of Internal Medicine, Respiratory Medicine and Ventilatory Support, Medical Mission Hospital, Central Clinic Würzburg, Würzburg, Germany

**Keywords:** Left heart disease, Post-capillary pulmonary hypertension, Guidelines

## Abstract

**Background:**

In 2022, the definition of pulmonary hypertension (PH) in the presence of left heart disease was updated according to the new joint guidelines of the European Society of Cardiology (ESC) and the European Respiratory Society (ERS). The impact of the new ESC/ERS definition on the prevalence of post-capillary PH (pc-PH) and its subgroups of isolated post-capillary (Ipc-PH) and combined pre- and post-capillary PH (Cpc-PH) in patients with left heart disease is unclear.

**Methods:**

We retrospectively identified *N* = 242 patients with left heart disease with available data on right heart catheterisation (RHC) and cardiac magnetic resonance imaging (CMR). The proportion of pc-PH and its subgroups was calculated according to the old and new ESC/ERS PH definition. As the old definition did not allow the exact allocation of all patients with pc-PH into a respective subgroup, unclassifiable patients (Upc-PH) were regarded separately.

**Results:**

Seventy-six out of 242 patients had pc-PH according to the new ESC/ERS definitions, with 72 of these patients also meeting the criteria of the old definition. Using the old definition, 50 patients were diagnosed with Ipc-PH, 4 with Cpc-PH, and 18 with Upc-PH. Applying the new definition, Ipc-PH was diagnosed in 35 patients (4 newly), and Cpc-PH in 41 patients. No CMR parameter allowed differentiating between Ipc-PH and Cpc-PH, regardless of which guideline version was used.

**Conclusion:**

Applying the new ESC/ERS 2022 guideline definitions mildly increased the proportion of patients diagnosed with pc-PH (+ 5.5%) but markedly increased Cpc-PH diagnoses. This effect was driven by the allocation of patients with formerly unclassifiable forms of post-capillary PH to the Cpc-PH subgroup and a significant shift of patients from the Ipc-PH to the Cpc-PH subgroup.

**Graphical abstract:**

**Figure:**
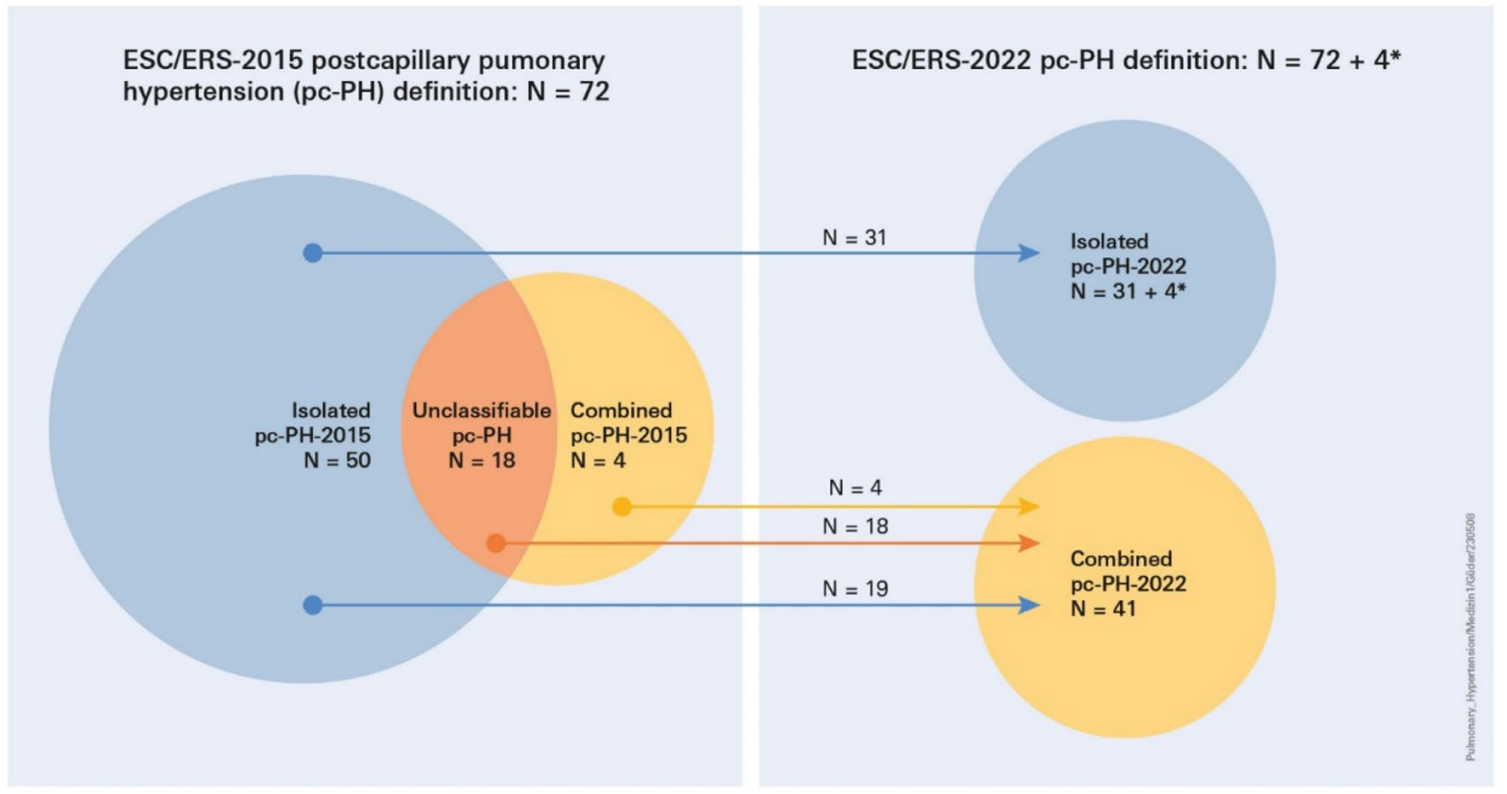
Distribution of post-capillary pulmonary hypertension (pc-PH) subgroups according to the European Society of Cardiology/European Respiratory Society (ESC/ERS) PH guidelines from 2015 and 2022 in N = 242 patients with left heart disease

## Introduction

Recently, an updated definition of pulmonary hypertension (PH) was published by the new joint guidelines of the European Society of Cardiology (ESC) and the European Respiratory Society (ERS) [1]. Compared to the previous version of the guidelines [2], a lower threshold of > 20 mmHg instead of ≥ 25 mmHg mean pulmonary artery pressure (PAP) was introduced for defining PH to identify patients at risk earlier [1]. Further, an increase in pulmonary vascular resistance (PVR) above 2 Wood units (WU) was added for diagnosing pre-capillary PH [1]. The threshold discriminating between pre- and post-capillary PH remained unchanged at a mean pulmonary artery wedge pressure (PAWP) level of > 15 mmHg (Table 1) [1].

**Table 1:** Definition of pulmonary hypertension and subtypes

	PH guidelines (ESC/ERS) 2015 [2]	PH guidelines (ESC/ERS) 2022 [1]
Pre-capillary PH	mPAP > 25 mmHg mPAWP ≤ 15 mmHg	mPAP > 20 mmHg mPAWP ≤ 15 mmHg PVR > 2 WU
Post-capillary PH	mPAP ≥ 25 mmHg mPAWP > 15 mmHg	mPAP > 20 mmHg mPAWP > 15 mmHg
Ipc-PH	mPAP ≥ 25 mmHg mPAWP > 15 mmHg DPG < 7 **and/or** PVR ≤ 3 WU	mPAP > 20 mmHg mPAWP > 15 mmHg PVR ≤ 2 WU
Cpc-PH	mPAP ≥ 25 mmHg mPAWP > 15 mmHg DPG ≥ 7 and/or PVR > 3 WU	mPAP > 20 mmHg mPAWP > 15 mmHg PVR > 2 WU

*Cpc-PH* combined post- and pre-capillary pulmonary hypertension, *DPG* diastolic pressure gradient, (diastolic pulmonary artery pressure–mPAWP), *Ipc-PH* isolated post-capillary pulmonary hypertension, *mPAP* mean pulmonary arterial pressure, *mPAWP* mean pulmonary arterial wedge pressure, *PH* pulmonary hypertension, *PVR* pulmonary vascular resistance, *WU* Wood units

Post-capillary PH, the most common form of PH, is primarily seen in conditions presenting with left heart disease, including heart failure (HF) or valvular heart disease (VHD) [3]. This subgroup can be divided into isolated post-capillary PH (Ipc-PH) and combined post- and pre-capillary PH (Cpc-PH) [1–3]. Ipc-PH describes a phenotype with predominant post-capillary PH, while Cpc-PH shares both post- and pre-capillary PH features [2, 4].

**Fig. 1:**
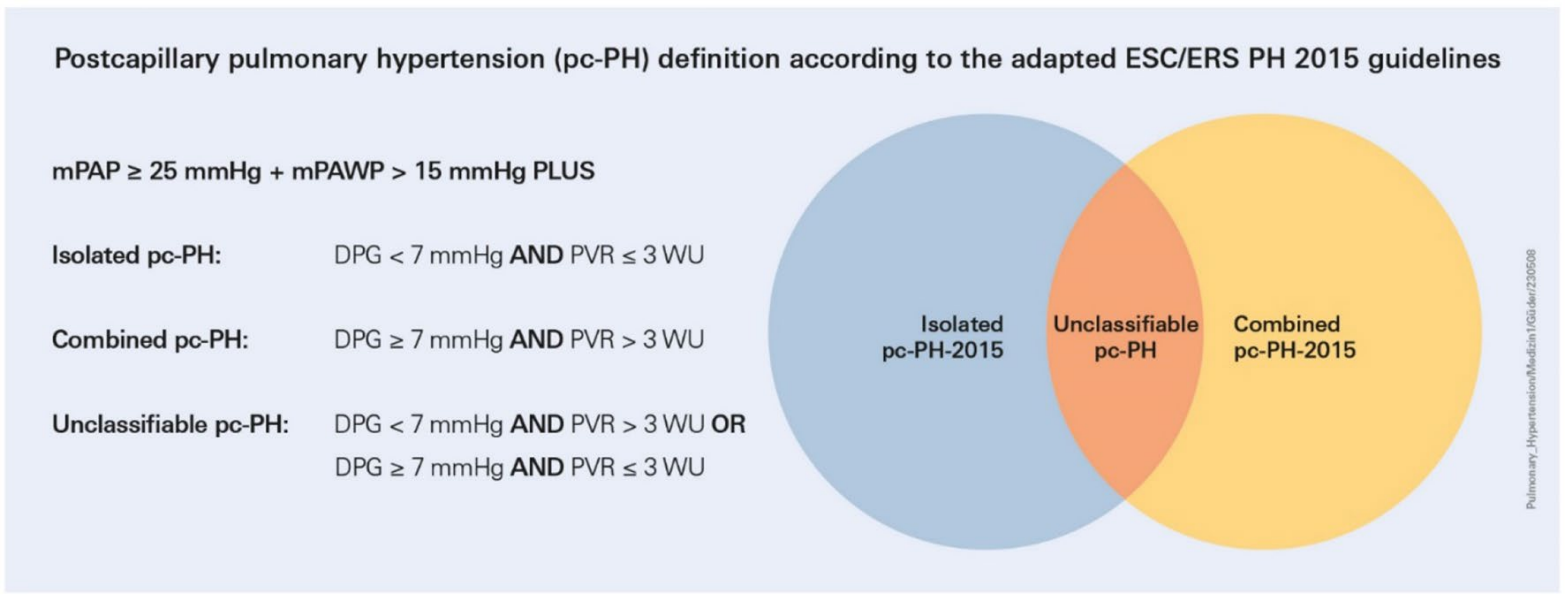
Adapted ESC/ERS 2015 post-capillary pulmonary hypertension (pc-PH) definition. *DPG* diastolic pressure gradient, *mPAP* mean pulmonary artery pressure, *mPAWP* mean pulmonary artery wedge pressure, *PVR* pulmonary vascular resistance, *WU* wood units

Besides an mPAP ≥ 25 mmHg plus mean PAWP > 15 mmHg, a “diastolic pressure gradient (DPG) < 7 mmHg and/or a PVR ≤ 3 WU” were required in the former PH guidelines for diagnosing Ipc-PH and a “DPG ≥ 7 mmHg and/or a PVR > 3 WU” for diagnosing Cpc-PH (Table 1) [2, 4]. The “and/or filter” of the former Ipc-PH/Cpc-PH definitions was criticised as profiles consisting of “DPG ≥ 7 mmHg or PVR > 3 WU” could not be assigned clearly to either group (“unclassifiable pc-PH”; Fig. 1) [5, 6]. The flexibility of the definition was somehow intended because the group of patients with “DPG ≥ 7 mmHg or PVR > 3 WU” was regarded as an intermediate-risk group, as low risk was assumed in Ipc-PH (i.e., DPG < 7 mmHg and PVR ≤ 3 WU), and high risk in Cpc-PH (i.e., DPG ≥ 7 mmHg and PVR > 3 WU) [6].

In the current post-capillary PH definitions, the threshold for PVR was reduced to 2 WU, and DPG was discarded entirely, as its predictive potency was inconsistent [7, 8]. Post-capillary PH was defined as mean PAP > 20 mmHg and mean PAWP > 15 mmHg. Accordingly, Ipc-PH was defined as post-capillary PH and PVR ≤ 2 WU, and Cpc-PH as post-capillary PH and PVR > 2 WU (Table 1) [1].

The impact of the modified definitions on the frequency of post-capillary PH and its subtypes is unclear. We compared the prevalence rates of post-capillary PH, Ipc-PH, and Cpc-PH according to the 2015 and 2022 PH guideline definitions in patients with left heart disease [1, 2]. We further investigated whether the changes in the distribution of post-capillary PH subtypes brought upon by the new definition may originate from differences in hemodynamic and morphological characteristics assessed during right heart catheterisation (RHC) and cardiac magnetic resonance imaging (CMR).

## Methods

### Study design and patient selection

We identified patients with left heart disease (heart failure of any kind, valvular heart disease, or both) who had been treated in the Department of Internal Medicine at the University Hospital of Würzburg and had undergone RHC and CMR with the Data Ware House system. This electronic storage program connects patient data from different sources, such as discharge letters, International Classification of Diseases codes, diagnostic reports, and procedure codes [9]. The data protection officer controlling the data transfer via the Data Ware House at our institution approved this study’s data extraction, and the local Ethics Committee waived ethical approval. The study was conducted in compliance with the Declaration of Helsinki.

Interrogating the DWH, we identified 293 consecutive patients with information available on both RHC and CMR between January 2016 and January 2022. In 27 patients, pulmonary disease was predominant, and comorbid left heart disease could be excluded. These patients were not included in the analysis. Post-capillary PH, according to the new guideline definition, was diagnosed in 100 out of the remaining 266 patients (37.6%). Of those, 24 patients had to be excluded from analysis because cardiac CMR was older than 2 weeks (*N* = 9), information on cardiac output (CO) according to the thermodilution method [10] was missing (*N* = 12), or cardiac shunts were detected precluding the reliability of CO measurement (*N* = 3). Accordingly, the analysis covers 76 patients with post-capillary PH as defined by the new PH guidelines.

Clinical data such as comorbidities, medication, electrocardiogram, cardiac imaging data, coronary angiography, and RHC were collected from medical records. Transthoracic echocardiography was performed according to practice guidelines [11] as part of the clinical routine either during the same hospitalisation or before hospitalisation as part of an outpatient visit. The median time difference between echocardiography and RHC was 1 day (quartiles 0, 4 days). CMR was performed on either a 1.5 T Achieva or a 3.0 T Achieva D scanner (Philips Healthcare, Best, The Netherlands) [12]. The median time difference between CMR and RHC was 4 days (quartiles 1, 6 days). Standard CMR parameters were assessed; right ventricular-pulmonary arterial coupling (RV-PA) was calculated using the ratio between RV stroke volume (SV) and RV end-systolic volume ratio (ESV) as described before [13, 14].

RHC was performed according to standard recommendations [15], either alone or combined with coronary angiography using an Edwards Lifesciences Vigilance II™ monitor or the Schwarzer Cardiotek Evolution system. Cardiac output was measured according to the thermodilution method [10]. The ABL80 FLEX CO-OX blood gas analyser was used to measure haemoglobin levels and oxygen saturation of mixed venous blood (PA-SO_2_). Arterial oxygen saturation (SaO_2_) was derived from finger pulse oximetry or measured invasively in patients with additional arterial catheterisation. The diastolic pressure gradient (DPG) was calculated as diastolic pulmonary artery pressure (dPAP) minus mean PAWP [2]. The transpulmonary pressure gradient (TPG) was calculated as mean PAP minus mean PAWP [2]. The formula of Dubois & Dubois was applied to calculate the body surface area (BSA) [16]. Data from RHC (hemodynamics and pressure tracings) were double-checked and entered manually by two cardiologists (GG and TR).

### Definition of heart failure

All patients included had signs or symptoms of heart failure. Heart failure (HF) was defined according to the ESC HF guidelines as HF with reduced ejection fraction (HFrEF, left ventricular ejection fraction, LVEF ≤ 40%) and HF with mildly reduced ejection fraction (HFmrEF, LVEF 40–49%) [17]. As all patients included had a mean PAWP > 15 mmHg, HF with preserved ejection fraction (HFpEF) was defined according to the consensus recommendations of the ESC Heart Failure Association as LVEF ≥ 50% in the absence of significant (°III) left-sided valvular heart disease [18].

### Definition of post-capillary PH

The definitions of post-capillary PH and its subgroups according to the 2015 and 2022 ESC/ERS guidelines are shown in Table 1 [1, 2].

In 2015, post-capillary PH was defined as a mean PAP ≥ 25 mmHg and mean PAWP > 15 mmHg [2]. Ipc-PH was diagnosed if the diastolic pressure gradient (DPG) was < 7 mmHg and/or the PVR was ≤ 3 WU. Cpc-PH was diagnosed if the DPG was ≥ 7 mmHg and/or the PVR was > 3 WU. Due to the “and/or filter” of the 2015 definition, patients with a post-capillary PH with a “DPG ≥ 7 mmHg and PVR ≤ 3 WU” and patients with a “DPG < 7 mmHg and PVR > 3 WU” could not be assigned clearly (Table 1) [2, 5, 6]. We, therefore, analysed these patients in a separate category (i.e., unclassifiable post-capillary PH or Upc-PH, Fig. 1) and elaborated the shift to the respective group (Ipc-PH or Cpc-PH) according to the 2022 PH guideline definition.

In 2022, post-capillary PH was defined as mean PAP > 20 mmHg and mean PAWP > 15 mmHg, Ipc-PH was diagnosed if PVR was ≤ 2 WU and Cpc-PH if PVR was > 2 WU (Table 1) [1].

### Data analysis

Data are shown as count (per cent) or median (quartiles). Group comparisons were performed for nominal and ordinal parameters using exact Fisher’s or Chi-square tests and for metric parameters using Mann–Whitney *U* test or Kruskal Wallis test. A significant group difference was assumed for all test procedures at a (two-sided) *p* value of < 0.05. All statistical analyses were performed using IBM SPSS Statistics for Windows Version 28.

## Results

In total, 100 out of 266 (38%) patients with left heart disease were diagnosed with post-capillary PH according to the 2022 ESC/ERS PH guidelines definition. However, 24 patients had to be excluded due to missing values. Therefore, the current analysis reports on 76 patients with post-capillary PH according to the 2022 PH guideline definition. Of those, 72 patients were diagnosed with post-capillary PH according to the 2015 PH guideline definition (mean PAP ≥ 25 mmHg, mean PAWP > 15 mmHg) and additional 4 patients (+ 5.5%) if the new definition (mean PAP > 20 mmHg; mean PAWP > 15 mmHg) was applied.

The majority of patients with post-capillary PH were men (*N* = 56 of *N* = 76; 74%), with a mean age of 64 ± 15 years. The most common origin of left heart disease was HFrEF (*N* = 49; 64%), followed by valvular heart disease °III (*N* = 29; 38%), and HFpEF (*N* = 10; 13.2%); HFmrEF was diagnosed in 6.5% (*N* = 5). The most common high-grade valvular disease was aortic valve stenosis (AVS; *N* = 14 of *N* = 29; 48%). In four patients, combined high-grade valvular disease was prevalent (two mitral valve regurgitation, one high-grade tricuspid valve regurgitation, and one pulmonary valve stenosis after Ross procedure). High-grade mitral valve regurgitation was the second most common valvular heart disease (*N* = 11 out of *N* = 29; 38%) followed by aortic valve regurgitation (*N* = 2 out of 29; 6.8%). The other two patients had mitral valve stenosis and high-grade TR combined with HFpEF.

### Application of the former guideline definition.

Applying the old definitions, 50 out of 72 patients with post-capillary PH had Ipc-PH, 4 had Cpc-PH, and 18 were categorised as Upc-PH.

Patients in the Ipc-PH group had higher levels of LVEF, tricuspid annular plane systolic excursion (TAPSE), cardiac output (CO), cardiac index (CI) and mixed oxygen saturation (PA-SO_2_) than patients with Upc-PH (Table 2, *p* for all < 0.05). By contrast, mean PAP, DPG, TPG, and PVR were significantly lower (Table 2, *p* for all < 0.001). Associations for hemodynamic measures were similar if Ipc-PH was compared to the Cpc-PH group (*p* for all < 0.05). By contrast, the Upc-PH group differed from the Cpc-PH group only in BMI and DPG levels; all other hemodynamic measures were comparable.

**Table 2:** Baseline characteristics

	2015 PH guideline definition [2]							2022 PH guideline definition [1]				
	*N*	Ipc-PH *N* = 50	*N*	Upc-PH *N* = 18	*N*	Cpc-PH *N* = 4	*p*	*N*	Ipc-PH *N* = 35	N	Cpc-PH *N* = 41	*p*
Age, years	50	67 (57; 75)	18	72 (61; 78)	4	54 (41; 70)	0.15	35	66 (54; 76)	41	67 (57; 76)	0.73
Men, *N* (%)	50	39 (78%)	18	12 (67%)	4	3 (75%)	0.64	35	28 (80%)	41	28 (68%)	0.30
BMI, kg/m^2^	50	27.2 (24.3; 30.7)	18	27.1 (23.6; 28)	4	32.6 (29.6; 36)^#§^	**0.035**	35	27 (24.0; 30.9)	41	27.8 (24.2; 30.3)	0.75
HFrEF, *N* (%)	50	29 (58%)	18	15 (83%)	4	2 (50%)	0.13	35	21 (60%)	41	28 (68%)	0.48
HFpEF, *N* (%)	50	7 (14%)	18	2 (11%)	4	1 (25%)	0.77	35	5 (14%)	41	5 (12%)	1.00
VHD °III, *N* (%)	50	20 (40%)	18	7 (39%)	4	1 (25%)	0.84	35	10 (29%)	41	19 (46%)	0.16
NYHA III/IV, *N* %)	50	31 (62%)	18	10 (56%)	4	2 (50%)	0.82	35	19 (54%)	41	26 (65%)	0.35
GFR, ml/min/1,73qm	49	60 (44; 78)	18	71 (50; 82)	4	77 (73; 84)	0.32	34	66 (48; 78)	41	70 (51; 83)	0.62
NTproBNP, pg/ml	42	3569 (1044; 9015)	9	7069 (5419; 20,880)	4	2439 (445; 6594)	0.18	28	2570 (1110; 9365)	30	5728 (2171; 8648)	0.24
Echocardiography												
LVEF, %	49	43 (24; 53)	18	27 (23; 34)*	4	46 (23; 52)	0.07	34	39 (19; 53)	41	33 (24; 51)	0.93
*E*/*e’*	35	15 (11; 22)	13	22 (16; 27)	4	19 (10; 31)	0.20	25	14 (12; 18)	30	21 (12; 27)	0.08
TAPSE, mm	50	17 (15; 21)	18	14 (12; 19)*	4	15 (13; 16)	**0.033**	35	18 (15; 21)	41	15 (12; 19)	**0.044**
TR-Vmax, m/s	46	2.9 (2.6; 3.3)	17	3.2 (2.9; 3.7)	4	3.7 (2.8; 4.2)	0.11	32	2.8 (2.4; 3.3)	39	3.2 (2.7; 3.7)	**0.026**
TR-Pmax, mmHg	46	35 (26; 44)	18	44 (33; 55)	4	54 (31; 69)	**0.06**	32	30 (23; 43)	40	41 (32; 55)	**0.014**
RHC												
mPAWP, mmHg	50	23 (20; 27)	18	24 (20; 28)	4	21 (19; 27)	0.84	35	23 (19; 26)	41	23 (20; 28)	0.69
mPAP, mmHg	50	33 (29; 37)	18	39 (34; 49)**	4	46 (40; 48)^##^	** < 0.001**	35	29 (25; 34)	41	39 (33; 42)	** < 0.001**
mRAP, mmHg	49	11 (8; 14)	17	10 (9; 13)	4	16 (6; 23)	0.55	34	10 (7; 13)	40	11 (8; 14)	0.47
DPG, mmHg	50	-2.0 (-5.0; 1.0)	18	3.5 (1.5; 5.3)**	4	10 (7; 13)^##§^	** < 0.001**	35	-4.0 (-6.0; -1.0)	41	3.0 (-1.0; 4.5)	** < 0.001**
TPG, mmHg	50	9 (7; 13)	18	16 (14; 19)**	4	23 (17; 28)^##^	** < 0.001**	35	7 (5; 9)	41	14 (13; 18)	** < 0.001**
PVR, WU	50	1.7 (1.3; 2.4)	18	4.2 (3.2; 5.5)**	4	5.8 (4.4; 7.5)^##^	** < 0.001**	35	1.4 (0.9; 1.6)	41	2.9 (2.5; 4.6)	** < 0.001**
CO, L/min	50	5.4 (4.9; 6.4)	18	3.8 (3.3; 5.0)**	4	3.8 (3.3; 4.4)^#^	** < 0.001**	35	5.5 (4.8; 6.6)	41	4.5 (3.4; 5.4)	**0.001**
CI, L/min/m^2^	50	2.8 (2.4; 3.1)	18	2.1 (1.9; 2.6)**	4	1.9 (0.8; 2.4)^#^	** < 0.001**	35	2.7 (2.4; 3.2)	41	2.4 (2.0; 2.8)	**0.001**
PA-SO_2_, %	50	63 (58; 68)	18	59 (51; 64)*	4	50 (46; 61)^#^	**0.009**	35	65 (60; 70)	41	60 (51; 64)	** < 0.001**
AO-SO_2_, %	50	96 (95; 98)	18	97 (93; 98)	4	92 (88; 96)	0.15	35	96 (95; 98)	41	96 (93; 98)	0.22
CMR												
LA-area mm^2^	50	33 (27; 39)	18	33 (29; 37)	4	30 (15; 50)	0. 91	34	32 (26; 39)	41	33 (29; 38)	0.44
RA-area, mm^2^	50	28 (22; 32)	18	26 (19; 31)	4	24 (20; 28)	0.30	34	29 (21; 33)	41	27 (21; 31)	0.44
RA/LA-Ratio	50	0.82 (0.73; 0.94)	18	0.70 (0.62; 0.86)	4	0.93(0.45; 1.81)	0.23	34	0.85 (0.72; 0.97)	41	0.76 (0.62; 0.94)	0.14
LVEDD, mm	50	65 (55; 72)	18	66 (57; 72)	4	69 (48; 78)	0. 87	34	64 (55; 71)	41	66 (57; 74)	0.55
RVEDD, mm	50	35 (29; 38)	18	32 (28; 40)	4	35 (34; 40)	0.84	34	35 (29; 39)	41	35 (30; 39)	0.88
LVEF, %	50	36 (24; 50)	18	28 (24; 34)	4	42 (30; 60)	0.14	34	35 (24; 55)	41	30 (24; 45)	0.35
RVEF, %	47	47 (39; 59)	18	44 (39; 53)	4	35 (25; 47)	0.21	34	48 (37; 61)	41	45(38; 55)	0.47
LV-SVI, ml/m^2^	50	41 (33; 49)	18	38 (34; 45)	4	55 (33; 68)	0.42	32	40 (35; 48)	40	42 (34; 50)	0.60
RV-SVI, ml/m^2^	50	39 (31; 50)	18	38 (31; 47)	3	30 (22; 51)	0.52	34	41 (32; 51)	40	38 (30; 47)	0.22
LV-EDV, ml	50	230 (159; 317)	18	244 (188; 285)	4	338 (145; 370)	0.61	34	227 (157; 308)	41	253 (181; 339)	0.37
RV-EDV, ml	50	175 (136; 219)	18	159 (119; 253)	4	236 (159; 365)	0.29	34	186 (144; 232)	41	175 (128; 235)	0.73
LV-ESV, ml	50	144 (77; 245)	18	173 (115; 205)	4	197 (66; 261)	0.67	34	144 (77; 237)	41	169 (111; 240)	0.42
RV-ESV, ml	50	91 (61; 116)	18	90 (47; 157)	4	147 (97; 255)	0.21	34	88 (58; 133)	41	96 (64; 139)	0.69
RV SV/ESV	50	0.88 (0.62; 1.41)	18	0.78 (0.63; 1.11)	4	0.55 (0.33; 0.90)	0.22	34	0.90 (0.59; 1.51)	41	0.83 (0.61; 1.18)	0.49

Significant values are in bold

Values are total numbers (and percentage of *n*) or median (25th–75th percentile). *p* values refer to Fisher’s exact test, Chi-Square test, Mann–Whitney *U*-test, or Kruskal–Wallis *H*-Test as appropriate. Further group comparisons within the 2015 group were performed between: (a) Ipc-PH vs Upc-PH, (marked with an asterisk (*) for *p* values < 0.05 and with two asterisks (**) for *p* values < 0.001), (b) Ipc-PH and Cpc-PH (marked with a hashtag (^#^) for *p* values < 0.05 and with two hashtags (^§§^) for *p* values < 0.001), (c) Upc-PH vs Cpc-PH (marked with a paragraph (^§^) for *p* values < 0.05 and with two paragraphs (^§§^) for *p* values < 0.001

*AO-SO*_*2*_ arterial oxygen saturation, *BMI* Body mass index, *CI* cardiac index, *CMR* cardiac magnetic resonance imaging, *CO* cardiac output, *Cpc-PH* combined post- and pre-capillary pulmonary hypertension, *DPG* diastolic pressure gradient (diastolic pulmonary artery pressure minus mean PAWP), *GFR* glomerular filtration rate, *HFpEF* heart failure with preserved ejection fraction, *HFrEF* heart failure with reduced ejection fraction, *Ipc-PH* isolated post-capillary pulmonary hypertension, *LA* left atrium, *LVEDD* left ventricular end-diastolic diameter, *LV-EDV* LV end-diastolic volume, *LVEF* LV ejection fraction, *LV-ESV* LV end-systolic volume, *mPAP* mean pulmonary artery pressure, *mPAWP* mean pulmonary artery wedge pressure, *mRAP* mean right atrial pressure, *NT-proBNP* N-terminal-prohormone of brain natriuretic peptide, *NYHA* New York Heart association class, *PA-SO2* mixed oxygen saturation in the pulmonary artery, *RA* right atrium, *RHC* right heart catheter, *RVEDD* right ventricular end-diastolic diameter, *RVEDV* RV end-diastolic volume, *RVEF* RV ejection fraction, *RV-ESV* RV end-systolic volume, *RV-SV/ESV* index of RV stroke volume to end-systolic volume, *PVR* pulmonary vascular resistance, *SVI* stroke volume indexed by body surface are, *TAPSE* tricuspid annular plane systolic excursion, *TPG* transpulmonary pressure gradient (mPAP minus mPAWP), *TR-Pmax* peak pressure of tricuspid valve regurgitation, *TR-Vmax* peak velocity of tricuspid valve regurgitation, *Upc-PH* unclassifiable postcapillary pulmonary hypertension, *VHD* valvular heart disease

### Application of the new guideline definition.

If the 2022 ESC/ERS definitions were applied, *N* = 4 patients with a mean PAP > 20 mmHg and < 25 mmHg were additionally diagnosed with Ipc-PH. Three of these patients had a LVEF of ≤ 20%, and one exhibited a restrictive diastolic pattern. Otherwise, patients were comparable to patients within the Ipc-PH group but had lower BMI, mean PAWP, mPAP and TPG levels (data not shown).

All patients categorised into Cpc-PH or Upc-PH according to former guidelines were grouped into Cpc-PH when applying the new guidelines. By contrast, only 62% (31 out of 50) of patients categorised into Ipc-PH according to former guidelines re-entered the Ipc-PH group when applying the new guidelines. The remaining 38% (*N* = 19) switched to the Cpc-PH-2022 subgroup.

The 2022 PH definition showed group differences between Ipc-PH and Cpc-PH for the TAPSE and peak tricuspid regurgitation velocity/pressure. Otherwise, only the groups' hemodynamic parameters (mean PAP, DPG, TPG, PVR, CO, CI, PA-SO2) significantly differed.

All other variables were distributed similarly (Table 2). Of note, none of the parameters derived from CMR significantly differed between post-capillary PH subgroups, neither according to the 2015 nor the 2022 PH definition (Table 2).

## Discussion

Using a lower mPAP threshold for defining post-capillary PH in the new ESC/ERS PH guidelines increased the prevalence of post-capillary PH in patients with predominant left heart disease by only 5%. By contrast, the changes related to the post-capillary PH subgroups definition had more impact as the number of patients diagnosed with Ipc-PH decreased by one-third, and the number of Cpc-PH diagnoses increased tenfold.

In 2018, the 6^th^ World Symposium on Pulmonary Hypertension (WSPH) suggested a comprehensive revision of the PH definition [19, 20]. The ESC/ERS 2022 PH guideline committee followed two of three recommendations for the new definition of post-capillary PH and its subgroups. First, the cut-off for the definition of PH was reduced from 25 to 21 mmHg [1, 19]. The previous threshold for the PH definition (mean PAP ≥ 25 mmHg) was chosen arbitrarily. In contrast, the new threshold for the definition of PH (mean PAP > 20 mmHg) was endorsed as evidence-based [2, 19, 20]. A systematic review revealed that the normal range of the mean PAP in healthy individuals is around 14 ± 3 mmHg at rest for both men and women [21]. Accordingly, based on the upper limit of the normal definition (mean plus two times the standard deviation), the correct threshold for an elevated mean PAP would be > 20 mmHg [1, 19]. The authors emphasized that the threshold of ≥ 25 mmHg encouraging treatment initiation in pre-capillary PH remained unchanged, as therapeutic studies only used the former threshold so far while conceding that a lower cut-off might help identify affected individuals at an earlier stage [1].

Second, DPG was discarded as a criterion for post-capillary PH subgroup definition [1]. The relevance of using a DPG threshold of > 7 mmHg for the differentiation between post-capillary PH subgroups in left heart disease has been criticized repeatedly, as DPG was neither prognostically relevant nor diagnostically reliable. For instance, many patients with high or very high PVR exhibited low or negative DPG values [22, 23]. The removal of DPG from the guideline definition had the positive side effect that the “and/or filter” could be deleted. Accordingly, the discussion about the correct allocation of patients with intermediate-risk fulfilling the 2015 criteria for both post-capillary PH subgroups (Upc-PH) ended [6, 24].

By contrast, the suggestion of the WSPH 2018 task force to adopt a PVR threshold of 3 WU was not implemented [8]. As the upper limit of normal for PVR lies about 2 WU and the prognostic significance of PVR starts at values slightly above 2 WU in both patients with pre- and post-capillary PH, the ESC/ERS PH guideline committee followed the argument that evidence-based thresholds should be preferred in guideline definitions and used a cut-off > 2 instead of 3 WU for discriminating Cpc-PH from Ipc-PH, [25, 26]

In our cohort, lowering the threshold according to the new PH definition from a mean PAP of ≥ 25 to > 20 mmHg did not change the number of patients with Cpc-PH. Only *N* = 4 patients were newly diagnosed with post-capillary pH, and all of them had a PVR below two WU and were allocated into the Ipc-PH-2022 subgroup.

Discarding DPG as a criteria component discriminating post-capillary subgroups (Table 1) increased the number of Cpc-PH diagnoses by enabling conclusive subgroup-allocation of all patients with post-capillary PH. Per definition, patients with Upc-PH had either a PVR > 3 WU (*N* = 16) and were reclassified as Cpc-PH with the 2022 PH definition anyway or a DPG ≥ 7 mmHg (*N* = 3). As patients with a DPG ≥ 7 mmHg tended to have a PVR > 2 WU, taken together all patients within the Upc-PH were re-classified into the Cpc-PH 2022 subgroup.

The component of the ESC/ERS 2022 PH guideline definition with the highest impact on the change of post-capillary PH subgroup distribution was lowering the PVR threshold from > 3 to > 2 WU (Table 1).

A significant part (*N* = 19 of 50; 38%) of patients formerly classified as Ipc-PH were reclassified by the new definition as Cpc-PH. Of note, if the third WSPH 2018 task force recommendation, which advocates a PVR threshold of 3 instead of 2 WU, had been used for defining post-capillary PH subgroups, none of the patients within the Ipc-PH 2015 group would have switched to the Cpc-PH 2022 subgroup [27].

Despite all the changes, the new PH and PH subgroup definitions drew little attention in the cardiology community. Regardless of the prognostic impact of PH in left heart disease, therapeutic options to address PH are still missing [7, 25, 28, 29]. For patients with valvular heart disease, restoration of valvular function is paramount [30, 31]. PH therapy in HF with reduced ejection fraction failed to show any benefits, and some studies even showed detrimental effects [3]. Thus, routine use of PH medication in HFrEF is not recommended [4]. PH treatment was also tested in patients with PH and HFpEF without convincing results [28]. Recently, the soluble guanylate cyclase stimulator (sGC) Vericiguat effectively reduced the primary endpoint in patients with HFrEF, mainly by lowering the risk for HF hospitalisations [32]. And although sGC may exert multiple favourable effects in heart failure, vasodilation of the pulmonary arteries may be one target. Thus, the chapter on the potential benefits of PH treatment in left heart disease still is not fully closed [33]. As PH treatment usually targets the pre-capillary component of PH, PH therapy may be considered only in selected patients with left heart disease and combined post- and pre-capillary PH, preferably under the continuous supervision of specialised centres [1, 4, 34]. The correct identification of patients with Cpc-PH is, therefore, of particular interest [24].

PVR is regarded as the resistance present in the pre-capillary pulmonary arteries and is calculated as the difference between mean PAP and mean PAWP divided by CO [26]. Patients with left heart disease may easily exceed the lower threshold of a PVR > 2 WU due to reduced CO levels, indicating that even patients with no or only a modest pre-capillary PH component may be classified as Cpc-PH which may complicate the identification of patients with Cpc-PH with an actual pulmonary vascular disease component.

Further, none of the clinical variables investigated showed a difference between Ipc-PH and Cpc-PH when the new definition was applied. With the old guideline version, Cpc-PH patients tended to be more obese than patients with Ipc-PH but did not show any other differences. Of note, CMR was also not suited to discriminate between Ipc-PH and Cpc-PH; neither functional (LVEF, RVEF, SV/ESV ratio) nor morphological characteristics (diameters, areas, volumes of atria and ventricles) were different, neither with the 2015 nor with the 2022 definition. In transthoracic echocardiography, patients with Cpc-PH had lower TAPSE levels and higher peak pressure gradients of tricuspid valve regurgitation than patients with Ipc-PH. Still, the overlap range was too wide to define a proper cut-off for subgroup discrimination.

Thus, RHC is paramount for identifying patients with Cpc-PH, but its value in patients with left heart disease continuously decreases [35]. The question which patient will benefit from referral to specialised centres for therapy evaluation once the diagnosis of Cpc-PH is established also remains unclear. Recently, a non-evidence-based PVR threshold of > 5 WU was recommended [34]. This calls into question whether lowering the PVR threshold in the new PH guidelines is of any practical use in patients with left heart diseases.

### Limitations

Certain limitations must be considered. This is a retrospective single-centre study with no standardized mode of data collection and rather small numbers of patients, especially within the Cpc-PH-2015 subgroup. However, data were collected within routine clinical practice and represent real-world conditions. Further, the inclusion of patients with additional CMR data may have selected healthier than average patients, as patients’ selection is based on the CMR exclusion criteria (e.g., non-CMR compatible cardiac device therapy, severe dyspnoea precluding longer stay in a flat position, chronic kidney disease with a GFR < 30 ml/min/1.73m^2^, etc.). Still, CMR allowed proper characterisation of cardiac morphology and function and reliably verified no between-(sub)group differences. Lastly, the study’s central message, the increase in the number of patients with Cpc-PH using the new guideline definitions, was not affected by all these shortcomings.

## Conclusions

In conclusion, the new ESC/ERS 2022 PH guideline definitions only modestly increased the number of patients diagnosed with post-capillary PH, but markedly changed the distribution within its’ subgroups towards a higher proportion of patients with Cpc-PH. Using the new criteria, not only previously unclassified but also a substantial part of formerly Ipc-PH-classified patients entered the Cpc-PH subgroup, questioning whether the pre-capillary PH component is of clinical relevance in all patients with Cpc-PH. Thus, the search for an accurate definition of Cpc-PH in left heart disease is seemingly not over yet.
